# Evaluation of Hyperbaric Oxygen Treatment in Acute Traumatic Spinal Cord Injury in Rats Using Diffusion Tensor Imaging

**DOI:** 10.14336/AD.2017.0726

**Published:** 2018-06-01

**Authors:** Wenzhi Sun, Jiewen Tan, Zhuo Li, Shibao Lu, Man Li, Chao Kong, Yong Hai, Chunjin Gao, Xuehua Liu

**Affiliations:** ^1^Department of Orthopaedics, Beijing Xuanwu Hospital, Capital Medical University, Beijing 100020, China.; ^2^Department of Hyperbaric Oxygen, Sun Yat-Sen Memorial Hospital, Sun Yat-Sen University, Guangzhou, 510520, China.; ^3^Department of Hyperbaric Oxygen, Beijing Chaoyang Hospital, Capital Medical University, Beijing 100020, China.; ^4^Departments of Radiology, Beijing Chaoyang Hospital, Capital Medical University, Beijing 100020, China.; ^5^Department of Orthopaedics, Beijing Chaoyang Hospital, Capital Medical University, Beijing 100020, China.

**Keywords:** diffusion tensor imaging, hyperbaric oxygen, spinal cord injury

## Abstract

This study aimed to evaluate the therapeutic effect of hyperbaric oxygen (HBO) on acute spinal cord injury (SCI) by measuring the in vivo diffusion tensor imaging (DTI) parameters apparent diffusion coefficient (ADC) and fractional anisotropy (FA) and observing diffusion tensor tractography (DTT) of fiber bundle morphology. The rats were randomly divided into sham-operated (SH), SCI, and SCI and hyperbaric oxygen treatment (SCI + HBO) groups (n = 6 in each group). The Basso-Bettie-Bresnahan (BBB) score was used to evaluate motor function recovery, and DTI was performed on days 3, 7, 14, and 21 after surgery. BBB scores and FA values decreased significantly after SCI, while the two values significantly improved in the SCI + HBO group compared with the SCI group on days 7, 14, and 21. ADC increased significantly on days 14 and 21 postoperatively in the SCI group compared with the SH group but did not significantly differ between the SCI and SCI + HBO groups at any time point. BBB scores had the same variation trend with ADC values and FA values in all three groups. In the SH group, DTT showed a well-organized spinal cord, but the spinal cord showed interruptions at sites of injury after SCI. In conclusion, HBO promotes the recovery of neuronal function after SCI. Parameters of DTI, especially FA, can quantitatively evaluate the efficacy of HBO treatment in SCI, while DTT enables the visualization of the fiber tracking of spinal cord tracts.

Spinal cord injury (SCI), including both primary and secondary injuries, always leads to disastrous consequences. The primary injury, which is caused by the initial mechanical insult, cannot be controlled. Secondary injury includes hemorrhage, edema, and inflammation and also has a direct impact on clinical outcomes.

Hyperbaric oxygen (HBO) has gradually become indispensable in treating SCI; however, the underlying mechanisms are yet to be characterized. HBO treatment may mitigate secondary injury to SCI by inhibiting inflammatory responses, promoting neurological recovery, reducing SCI-induced edema in the spinal cord, stabilizing the barrier of blood-spinal cord, and so forth. At present, most evaluations of HBO treatment have been based on the histological and biochemical evaluation of excised spinal cord tissue. However, they have many drawbacks. First, they cannot provide important information about the spatial and temporal evolution of SCI because they show only a static view of the spinal cord tissue. Besides, they require the sacrifice of a large number of animals at different time points. Furthermore, histological evaluation cannot be conducted in humans. Consequently, a need exists for a noninvasive technique that can detect the progression of injury and evaluate the efficacy of HBO treatment in a single individual, and which can be translated to humans.

Currently, magnetic resonance imaging (MRI) is better than computed tomography examination in the noninvasive evaluation of spinal cord parenchyma after SCI and variation of signals, but conventional MRI lacks the sensitivity to detect and characterize spinal cord lesions [1]. A previous study found no significant difference in recovery rates between patients with and without intramedullary high signal intensity on preoperative T2-weighted images [2]. More importantly, changes in signal intensity in conventional MRI sequences are not related to a poor outcome of treatment or severity of SCI [3]. Sensitive methods are needed to reveal changes in the neurological structure so as to accurately and effectively assess the efficacy of therapeutic intervention after SCI.

Some reports have suggested that diffusion tensor imaging (DTI) has the potential to detect subtle pathology that may go undetected with conventional MRI techniques [4-6] and can predict the severity of SCI [7-11]. DTI is a more recent MRI-based technique that quantifies the diffusion of water molecules in directions parallel and transverse to the axis of neuronal axons. The most commonly used quantitative DTI parameters include fractional anisotropy (FA), which reflects the anisotropy of diffusion, and apparent diffusion coefficient (ADC), which reflects the magnitude of diffusion.

Although studies of DTI in SCI have been reported, this novels study evaluated the efficacy of HBO treatment in SCI-related changes of the thoracic spinal cord using DTI by measuring the changes in ADC and FA.

## MATERIALS AND METHODS

### Materials

Eighteen healthy adult male Sprague-Dawley (SD) rats, weighing 250-300 g, were provided by the Experimental Animal Center of the Academy of Beijing Military Medical Sciences (Beijing, China). The rats had access to food and water ad libitum and were housed in a room with a 12/12-h light/dark cycle. The rats were randomly divided into three groups with six rats in each group: SH, SCI, and SCI + HBO.

### SCI model

All experimental procedures were approved by the Committee for the Purpose of Control and Supervision of Experiments on Animals, Capital Medical University (Beijing, China), and carried out in accordance with relevant guidelines and regulations. The SCI rat model was established according to the method previously described by Basso et al. [12]. Briefly, after preparing the routine aseptic materials, SD rats were anesthetized with an intraperitoneal injection of 4% chloral hydrate at a dose of 100 mL/kg and placed in the prone position. Then, the dorsal skin of the rats was shaved, and an incision was made longitudinally to expose the spinous process and lamina. After sterilizing the T10 spinous process, it was removed together with the lamina to expose the spinal cord. The New York University Impactor (NYU impactor) was used to create an SCI with an energy of 25 gcm. The signs of the successful creation of the model were as follows: hemorrhage and edema were seen in the injured area; the dura became purple but remained intact; the wagging tail reflection emerged; the lower limbs retracted; the body fluttered; and flaccid paralysis was observed. The incision was closed in layers. After surgery, the rats were helped to empty their bladders twice a day until the micturition reflex recovered, and penicillin sodium was subcutaneously administered at 0.8 mg/g per day until the hematuria disappeared. The rats in the SH and SH + HBO groups also underwent a laminectomy, but their spinal cords were uninjured.

### HBO treatment

HBO treatment was conducted similarly to the method described in a previous study [13]. The rats in the SCI + HBO group were placed into an animal hyperbaric chamber (701 Space Research Institute, Beijing, China) immediately after surgery and received 1 h of HBO treatment in a 2.0-ATA chamber in 100% oxygen, and this was repeated daily at 4 p.m. Briefly, compressed air was supplied at a rate of 1 kg/cm^2^ per minute to 2.0 ATA/100% oxygen and maintained for 60 min. The chamber was flushed with 100% oxygen at a rate of 5 L per minute to avoid carbon dioxide accumulation. Decompression was performed at 0.2 kg/cm^2^ per minute. During HBO exposure, oxygen and carbon dioxide contents were continuously monitored and maintained at 98% or higher and at 0.03% or less, respectively. The rats in the sham and SCI groups were treated with normobaric air at 1.0 ATA in 21% oxygen.

### BBB score

Motor function recovery was evaluated using a modification of BBB locomotor rating scale [14] half an hour after HBO or normobaric air treatment on 3, 7, 14, and 21 days after surgery. Three well-trained individuals who were blinded to the study observed the behavior of rats for 5 min. The BBB score ranged from 0 to 21 according to the rating scale. Every rat had a BBB score of 21 before surgery. The BBB score would become 0 to 1 after a successful SCI.

### In vivo MRI and DTI

SD rats were anesthetized and mounted in the supine position within the scanner. All experiments were conducted on a 3.0-Tesla MR scanner (Discovery MR750; GE Healthcare, WI, USA) with a configuring dedicated animal coil. Conventional MRI and DTI scans were performed *in vivo* after evaluating the BBB score on days 3, 7, 14, and 21 after surgery.

For conventional MRI, a sagittal T1-weighted image (T1WI) and a sagittal T2-weighted image (T2WI) were obtained. An SE sequence with the following parameters was used to acquire anatomical images: sagittal T1WIs [TR/TE 9.6 ms/3.3 ms, 512 × 512 matrix, field of view (FOV) 16, slice thickness 1.5 mm] and sagittal T2WIs (TR/TE 1800 ms/108.7 ms, 256 × 256 matrix, FOV 8, slice thickness 1.4 mm).

DTI was performed in the axial view using an rFOV single-shot spin-echo planar imaging (ss-EPI) sequence with a *b* value = 800 s mm^-2^, TE 79.9 ms, TR 4000 ms, 98 × 48 matrix, FOV 8 × 4, slice thickness 3 mm, and 25 diffusion gradient directions.

### Image processing

Images were transferred to an independent workstation and analyzed by two radiologists who were blinded to the study. The ADC and FA values were measured using regions of interest (ROI). The ROIs were carefully placed so that they included both central gray and white matters. They were confirmed using the ADC and FA images, and care was taken to avoid the inclusion of cerebrospinal fluid. The FACT algorithm implemented in Volume-One software was used to perform DTT. The FA threshold was less than 0.2, and the stopping angle was more than 25°.

### Statistical analysis

All values are expressed as mean ± standard deviation. One-way analysis of variance was used to test the differences between different groups at the same time point. A *P* value less than 0.05 was considered statistically significant. The variation trends of DTI results and BBB scores were analyzed using the Mann-Kendall trend test to correlate DTI results with BBB scores. All analyses were carried out using SPSS version 19.0 (SPSS Inc., IL, USA).

**Figure 1.**
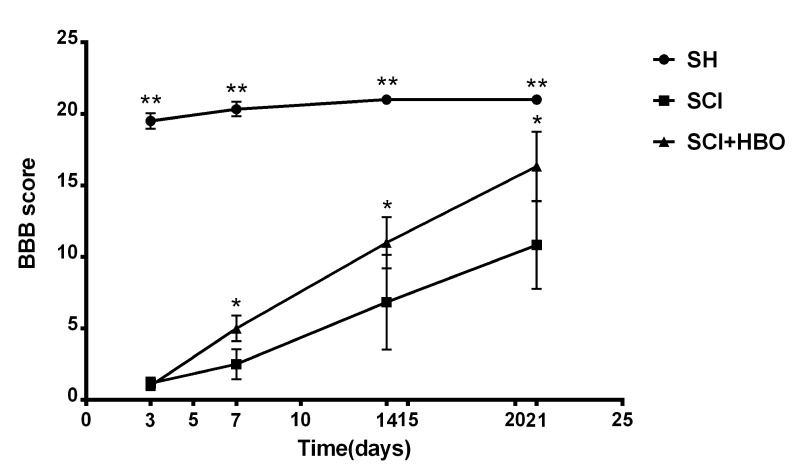
BBB scores for hind limb motor function in each group at different time points. The values are expressed as mean ± SD. **P < 0.001, *P < 0.01.

## RESULTS

### SCI model and BBB score

The rats in the SH group showed a BBB score greater than or equal to 20 points after surgery, while the rats in the SCI and SCI + HBO groups showed the complete paralysis of both lower extremities with a BBB score of 0-1. The differences were all statistically significant at all time points (*P* < 0.001). A significant gradual recovery was observed in the rats of SCI and SCI + HBO groups over time. The BBB scores were significantly improved in the SCI + HBO group compared with the SCI group on days 7, 14, and 21 after SCI (*P* < 0.01) (Fig. 1).

**Figure 2.**
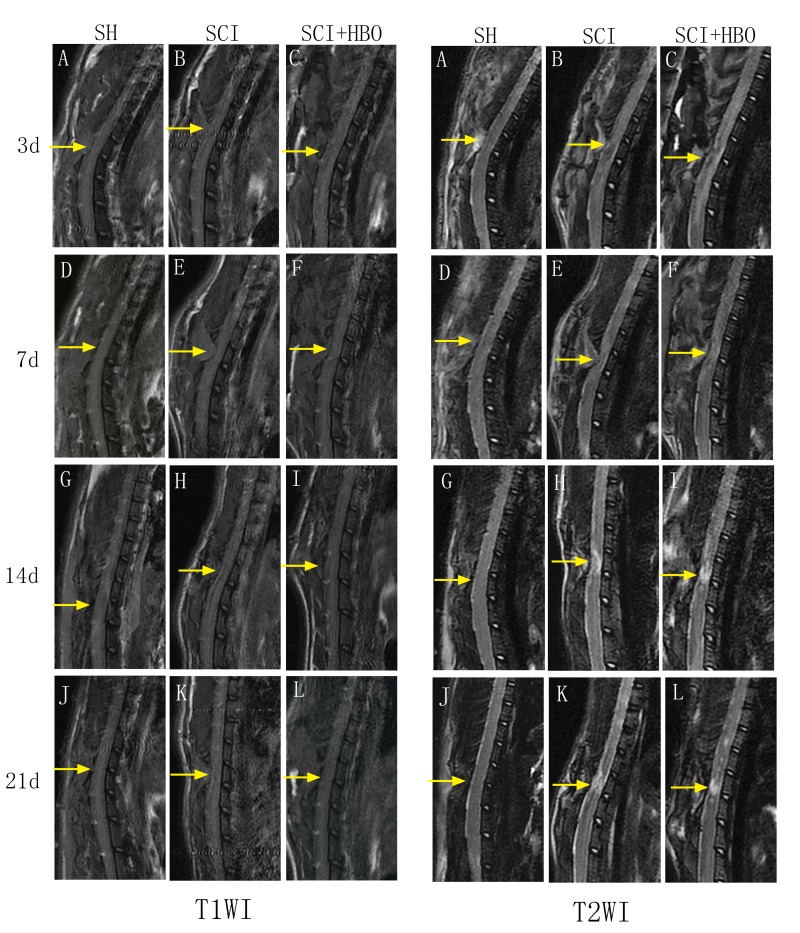
Conventional magnetic resonance images from the SH, SCI, and SCI + HBO groups at different time points. T1WI and T2WI in the SH group showed normal signal intensity at all time points (A, D, G, L). T1WI depicted less noticeable changes of the signal intensity after surgery, it still revealed the location of spinal cord lesions (A-L). T2WI of injured groups showed hypointense region within the central cord parenchyma surrounding a hyperintense region corresponding to the site of contusion on day 3 after SCI (B, C). Over time, the hypointense region faded away, while the hyperintense region became more noticeable (E, F, H, I, K, L).

### Conventional MRI

The rats in the SH group showed normal signal intensity at all time points and no evidence of spinal cord pathology. For rats in the SCI and SCI + HBO groups, although T1WI after SCI depicted less noticeable changes in signal intensity, it still revealed the location of spinal cord lesions. Meanwhile, sagittal T2WI on day 3 after SCI depicted a hypointense region within the central cord parenchyma surrounding a hyperintense region corresponding to the site of contusion. Over time, the hypointense region faded away, while the hyperintense region became more noticeable (Fig. 2).

### DTI

**Figure 3.**
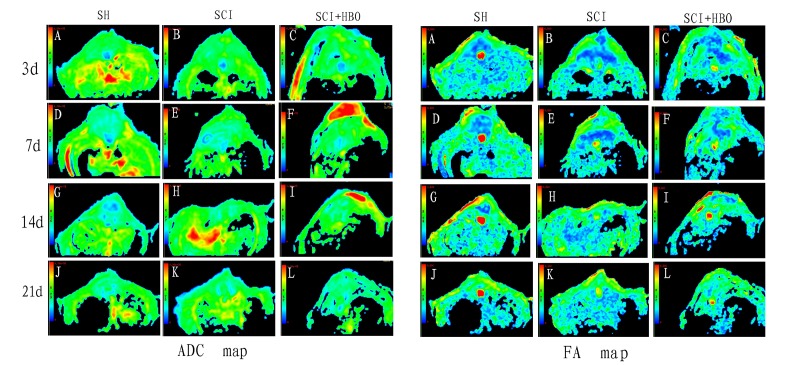
ADC and FA images in each group at different time points.

Maps of FA and ADC at all time points are shown in Figure 3. The part of blue region in the ADC map indicates the spinal cord, while the fading part of the blue region surrounding the spinal cord indicates the cerebrospinal fluid. In the ADC map, the differences among the three groups were not obvious. The red region in the FA map indicates the spinal cord, while the green region surrounding the red region indicates the cerebrospinal fluid. After SCI, the area of the red region decreased significantly. HBO intervention significantly increased the area of the red region.

**Figure 4.**
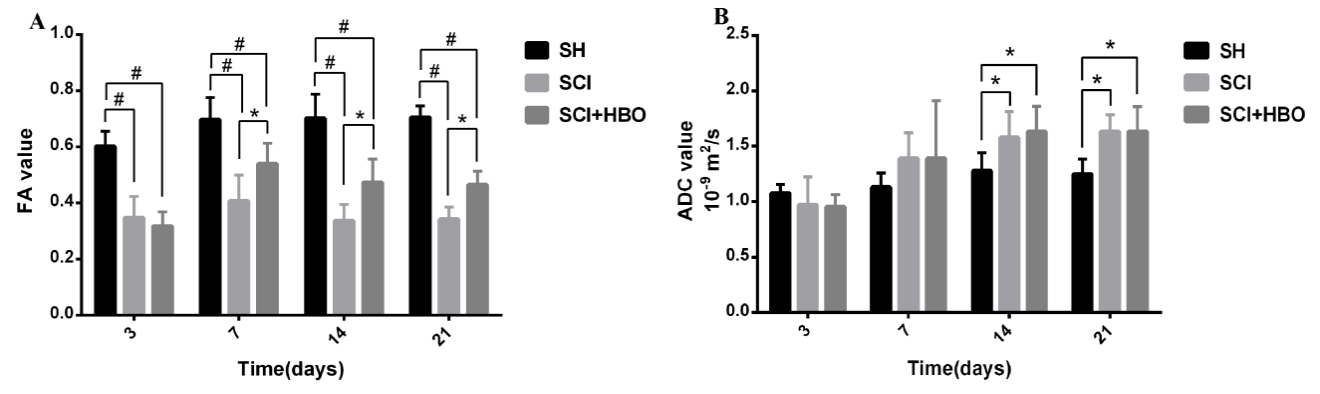
FA and ADC values of rats in the three groups. (A) FA values measured in the SH, SCI, SCI + HBO groups. (B) ADC values measured in the SH, SCI, and SCI + HBO groups. Values are expressed as mean ± SD. *P < 0.05, #*P* < 0.01.

**Figure 5.**
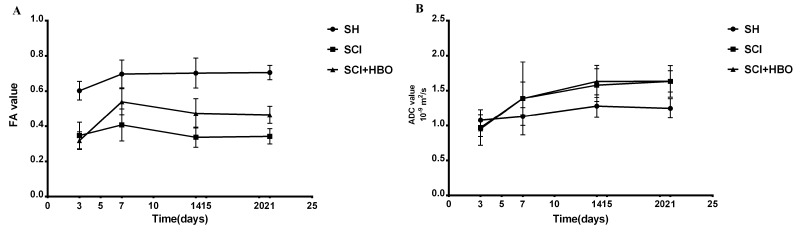
FA and ADC values of rats at different time points. (A) FA values measured in the SH, SCI, and SCI + HBO groups. (B) ADC values measured in the SH, SCI, and SCI + HBO groups.

FA and ADC values of rats in the three groups are listed in Tables 1 and 2. FA values decreased significantly in the SCI and SCI + HBO groups compared with those in the SH group at different time points after SCI (*P*< 0.01). Significant differences in FA values between the SCI and SCI + HBO groups were found only on days 7, 14, and 21 (*P*<0.05). ADC values increased significantly on days 14 and 21 postoperatively in the SCI group compared with those in the SH group (*P*<0.05), but no significant difference was found between the SCI and SCI + HBO groups at any time point (Fig. 4). The data also showed that FA values increased from day 3 to day 7 and then decreased, while a progressive increase was observed in ADC values in both the SCI and SCI + HBO groups (Fig. 5).

**Table 1:** FA values in SH, SCI, and SCI + HBO groups at different time points.

FA	SH	SCI	SCI + HBO
3 days	0.60 ± 0.05	0.35 ± 0.08	0.32 ± 0.05
7 days	0.70 ± 0.08	0.41 ± 0.09	0.54 ± 0.07
14 days	0.70 ± 0.09	0.34 ± 0.06	0.47 ± 0.08
21 days	0.71 ± 0.04	0.34 ± 0.04	0.46 ± 0.05

SH indicates the sham-operated group; SCI, spinal cord injury; HBO, hyperbaric oxygen; FA, fractional anisotropy.

### Variation trend of DTI results and BBB scores

**Figure 6.**
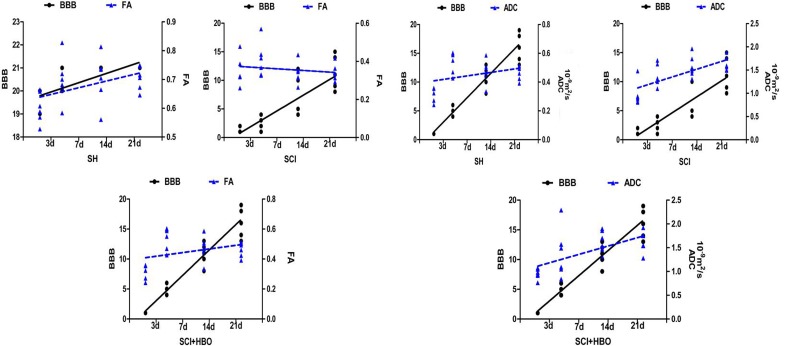
Variation trend of DTI results and BBB scores. (A) In all three groups (SH, SCI, and SCI + HBO), BBB scores and ADC values had the same variation trend after the Mann-Kendall trend test. (B) The same variation trend was found between the BBB score and the FA value.

It would be more convincing if a correlation analysis was made. The correlation analysis result was not reliable because this preliminary study had a small sample size. Therefore, the Mann-Kendall trend test was used to analyze the variation trend after normalization. The differences between different time points were not significant after normalization using the Mann-Kendall trend test, suggesting that BBB scores and ADC values had the same variation trend. Also, the same variation trend was found between the BBB score and the FA value (Fig. 6).

### DTT

**Figure 7.**
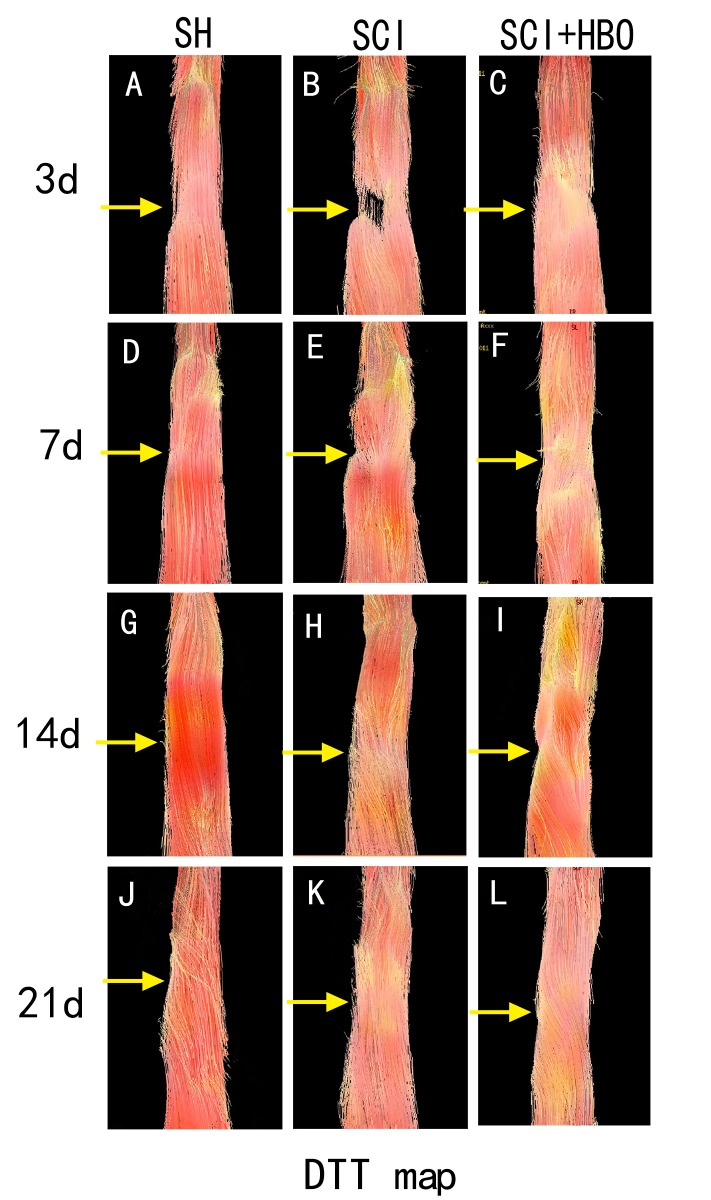
DTT of the three groups at different time points. DTT showed a continuous and intact spinal cord in uninjured rats (A, D, G, L); however, after SCI, the rats showed interrupted DTT (B, C). Over time, the spinal cord tracts in the injured groups gradually became continuous, with the tracts in the SCI + HBO group showing better continuity than the tracts in the SCI group at all time points (E, F, H, I, K, L).

The rats in the SH group showed continuous and intact DTT of the spinal cord, while the rats in the SCI and SCI + HBO groups showed interrupted DTT at the T10 vertebral level. Over time, the spinal cord tracts in the SCI and SCI + HBO groups gradually became continuous, with the tracts in the SCI + HBO group showing better continuity than the tracts in the SCI group at all time points (Fig. 7).

**Table 2:** ADC values in SH, SCI, and SCI + HBO groups at different time points.

ADC (10^-9^ m^2^/s)	SH	SCI	SCI + HBO
3 days	1.08 ± 0.08	0.97 ± 0.25	0.95 ± 0.11
7 days	1.13 ± 0.13	1.39 ± 0.23	1.39 ± 0.52
14 days	1.28 ± 0.16	1.58 ± 0.24	1.63 ± 0.23
21 days	1.25 ± 0.14	1.63 ± 0.15	1.63 ± 0.22

SH indicates the sham-operated group; SCI, spinal cord injury; HBO, hyperbaric oxygen; ADC, apparent diffusion coefficient.

The aforementioned results indicated that BBB scores and FA values decreased significantly after SCI and could also be significantly improved by HBO exposure. ADC values increased significantly on days 14 and 21 after SCI, but no obvious difference could be seen between the SCI + HBO and SCI groups at any time point. BBB scores had the same variation trend as ADC values and FA values in all three groups. In the SH group, DTT showed a well-organized spinal cord, but the spinal cord showed interruptions at sites of injury after SCI. HBO promoted the recovery of neuron function after SCI.

## DISCUSSION

Conventional MRI is routinely used for the qualitative assessment of the pathology of injured spinal cord. In the present study, T1WI after SCI depicted less noticeable changes in signal intensity, T2WI on day 14 after SCI did not show too many changes compared with images on day 21 after SCI, and T2WI in the SCI group depicted less difference with T2WI in the SCI + HBO group at the same time points. Therefore, conventional MRI can display SCI but cannot visualize more details. DTI with parameters such as ADC and FA can provide quantitative information about tissue microstructure. The use of DTI to detect subtle changes in brain tumors, diffuse axonal injury, multiple sclerosis, Alzheimer’s disease, ischemic stroke, and spinal cord has been reported [15]. ADC values represent the magnitude of diffusion, with high ADC values indicating the robust diffusive strength of extracellular water molecules. This information is derived from the DTI eigenvalues λ1, λ2, and λ3 that represent the magnitudes of diffusion along the three principal axes of the tissue structure [16]. FA is a dimensionless measure varying between 0 and 1, where 0 represents isotropic diffusion and 1 represents infinite anisotropy [17]. Unrestricted, isotropic diffusion is represented as a sphere in which all of the eigenvalues (λ) or diffusion coefficients are equal. Therefore, no directionality exists and FA is 0, for example, in pure water or cerebrospinal fluid [15]. Diffusion is often anisotropic in highly organized biological tissue [15] because axonal membranes and myelin sheaths present barriers to the motion of water molecules in directions not parallel to their own orientation.

Li et al. [11] evaluated the characteristics of DTI in the acute spinal cord of SD rats following a thoracic SCI using a 3-T scanner. They found that the ADC and FA values 3 days after moderate injury were 0.98 ± 0.19 and 0.39 ± 0.03, respectively. Rodrigo et al. [18] reported the *in vivo* quantitative MRI evaluation of moderate traumatic thoracic SCI in Long-Evans rats using a clinical 3-T scanner, and their ADC and FA values 7 days after SCI were 1.400 ± 0.169 and 0.276 ± 0.012, respectively. In the present study, ADC values at the T10 vertebral level were 0.97 ± 0.25 and 1.39 ± 0.23 and FA values were 0.35 ± 0.08 and 0.41 ± 0.09, respectively, 3 and 7 days after injury. Also, ADC values showed a progressive increase after injury. In the present study, FA values were maximal 7 days after injury.

The reasons for changes in ADC and FA values in the injured spinal cord are still uncertain. Tsuchiya et al. [19] considered that the increased ADC values indicated irreversible damage such as cystic necrosis, syrinx, and atrophy caused by myelomalacia. Song et al. [6] explained that the increase in ADC might be caused by a decrease in perfusion due to SCI, which might lead to ischemia and anoxemia, while cellular membrane injury could increase cellular membrane penetrability. The decreased FA values, in general, were attributed to the restricted diffusion of water molecules because of an increase in disorder after SCI. The presence of hemorrhage at the injury site might also prevent the free movement of water in the extracellular space [9]. Song et al. [20] observed decreased diffusion anisotropy in white matter tracts of the shiverer mouse CNS, which was due to the reduction in myelin. This study found that the FA value increased in the SCI + HBO group compared with the SCI group 7 days after surgery (*P* < 0.05). The reasons for this might be that HBO treatment could reduce edema, inflammatory response, and ischemia-reperfusion injury and promote the recovery of neuronal function [13, 21-23]. In conclusion, HBO treatment could help restore the original physiological state of the spinal cord as far as possible. Further, the efficacy of HBO treatment in SCI could be reflected by changes in the DTI parameters.

DTI parameters can provide functional predictions. In the clinic, FA of the whole cord correlated with the upper limb motor score as well as the ASIA grade [7]. Reduced FA and increased ADC values were seen in children with SCI compared with controls and showed good clinical correlation with ISNCSCI examinations [24]. Kim et al. [25] found that hyperacute (<3 h after injury) DTI of the lateral and ventral white matter at the injury epicenter was predictive of both electrophysiological and behavioral (locomotor) recovery at 4 weeks after injury, despite the presence of flaccid paralysis/spinal shock. Sundberg et al. [26] suggested that pathological changes in the spinal cord could be measured with DTI, and that they significantly affected the neurobehavioral outcome. The present study found that BBB scores had the same variation trend with ADC values and FA values in all three groups. Despite not being direct evidence, the statistical results showed that improved ADC values and FA values indicated the recovery of spinal cord.

DTT, as three-dimensional tract reconstruction, can be based on the DTI data, by which the orientation of nerve fibers can be followed to trace specific neural pathways [27]. It is useful for visually grading the neurological severity of SCI because it provides objective measures in terms of microstructural changes. Li et al. [11] found that the DTT of spinal cord could visualize the fiber tracking of spinal cord tracts, with the control rats exhibiting the well-organized fiber tracking of spinal cord tracts, while the injured rats with different severities of injury showed different characteristics at different time points. A basic imaging experiment also found that DTT could be useful for detecting the early changes associated with the compressed spinal cord of tiptoe-walking Yoshimura (twy) mice in the cervical ossification of posterior longitudinal ligament [28]. The applications of DTT were also reported to be used in patients with cervical spondylotic myelopathy [29, 30]. In this study, DTT showed a continuous and intact spinal cord in uninjured rats; however, after SCI, the rats showed interrupted DTT. In addition, fiber tracts of the rat spinal cord were much more organized after HBO treatment than those without HBO treatment at any time point.

This study had some limitations. First, the transmission radiofrequency field with 3-T imaging might not be as uniform as with 1.5 T [18]. Second, the quality of acquired images could not differentiate between white matter and gray matter tracts when an ROI was examined. Third, the histological examination was not performed. In addition, the quality of DTI of the thoracic spinal cord might be decreased due to the small diameter of the spinal cord, as well as respiratory and cardiac motion artifacts.

In conclusion, HBO treatment was shown to be beneficial for recovery after SCI. Values of ADC and FA measured by DTI could noninvasively and quantifiably evaluate the efficacy of HBO treatment in SCI. The DTT of spinal cord could visualize the fiber tracking of spinal cord tracts. Presently, more convincing clinical evidence is needed to confirm the effectiveness of HBO on SCI. Additionally, more scientific and reasonable HBO protocols for SCI such as pressure and treatment course also need to be explored. The results of this study recommended adopting DTI in the clinical observation of HBO on SCI. With the advantages of noninvasiveness, sensitivity, quantization, and objectivity, DTI might serve as a helpful and valuable evaluation index in future research.
